# Effects of perioperative enhanced recovery after surgery pathway management versus traditional management on the clinical outcomes of laparoscopic-assisted radical resection of distal gastric cancer: study protocol for a randomized controlled trial

**DOI:** 10.1186/s13063-020-04272-8

**Published:** 2020-05-01

**Authors:** Yulong Tian, Shougen Cao, Leping Li, Qingsi He, Lijian Xia, Lixin Jiang, Yinlu Ding, Xinjian Wang, Hao Wang, Weizheng Mao, Xizeng Hui, Yiran Shi, Huanhu Zhang, Xianqun Chu, Henrik Kehlet, Yanbing Zhou

**Affiliations:** 1 Department of Gastrointestinal Surgery, Affiliated Hospital of Qingdao University, No. 16 Jiangsu Road, Qingdao, China; 2 Department of Gastrointestinal Surgery, Shandong Provincial Hospital, Jinan, China; 3 Department of Gastrointestinal Surgery, Qilu Hospital of Shandong University, Jinan, China; 4 Department of Gastrointestinal Surgery, Qianfoshan Hospital of Shandong Province, Jinan, China; 5 Department of Gastrointestinal Surgery, Yantai Yuhuangding Hospital, Yantai, China; 6 Department of Gastrointestinal Surgery, Second Hospital of Shandong University, Jinan, China; 7 Department of Gastrointestinal Surgery, Weihai Central Hospital, Weihai, China; 8 Department of Gastrointestinal Surgery, Dongying People’s Hospital, Dongying, China; 9 Department of Gastrointestinal Surgery, Qingdao Municipal Hospital, Qingdao, China; 10 Department of Gastrointestinal Surgery, Rizhao People’s Hospital, Rizhao, China; 11 Department of Oncological Surgery, Weifang People’s Hospital, Weifang, China; 12 Department of Gastrointestinal Surgery, Weihai Municipal Hospital, Weihai, China; 13 Department of Gastrointestinal Surgery, Jining People’s Hospital, Jining, China; 14 Section of Surgical Pathophysiology 4074, Rigshospitalet Copenhagen University, Copenhagen, Denmark

**Keywords:** ERAS pathway, Traditional treatment, Gastric cancer, Laparoscopic distal gastrectomy, Clinical outcomes, Randomized controlled trial

## Abstract

**Background:**

The incidence of gastric cancer in East Asia is much higher than the international average. Therefore, improving the prognosis of patients and establishing effective clinical pathways are important topics for the prevention and treatment of gastric cancer. At present, the enhanced recovery after surgery (ERAS) pathway is widely used in the field of gastric surgery. Many randomized controlled trial (RCT) studies have proven that the ERAS regimen can improve the short-term clinical outcomes of patients with gastric cancer. However, a prospective study on the effect of the ERAS pathway on the prognosis of patients with gastric cancer has not yet been reported. This trial aims to confirm whether the ERAS pathway can improve the disease-free survival and overall survival of patients undergoing laparoscopic-assisted radical resection for distal gastric cancer.

**Methods/design:**

This study is a prospective, multicentre RCT. This experiment will consist of two groups – an experimental group and a control group – randomly divided in a 1:1 ratio. The perioperative period of the experimental group will be managed according to the ERAS pathway and that of the control group will be managed according to the traditional management mode. An estimated 400 patients will be enrolled. The main endpoint for comparison is the 3-year overall survival and disease-free survival between the two groups.

**Discussion:**

The results of this RCT should clarify whether the ERAS pathway is superior to traditional treatment on inflammatory indexes, short-term clinical outcome and survival for laparoscopic-assisted radical resection of distal gastric cancer. It is hoped that our data will provide evidence that the ERAS pathway improves survival in patients with gastric cancer.

**Trial registration:**

Chinese Clinical Trial Registry, CHiCTR1900022438. Registered on 11 April 2019.

## Introduction

### Background and rationale

Gastric cancer is a common malignant tumour with the third highest mortality rate worldwide. In 2018, more than 1.3 million new cases of gastric cancer were diagnosed and more than 780,000 deaths occurred [1]. Although the incidence of gastric cancer has decreased in the past 30 years, it is still very high in East Asia [2]. Indeed, there are more than 400,000 new cases of gastric cancer in China each year. The overall 5-year survival rate of these patients is about 30%, which is significantly lower than that in South Korea and Japan [3]. Overall, improving the comprehensive treatment effect of gastric cancer, ensuring the quality and safety of the perioperative period, improving the prognosis of these patients, and establishing an effective clinical pathway are important topics of research in China, and the primary goals are preventing and treating gastric cancer. At present, many treatment methods, such as surgery, radiotherapy, chemotherapy, targeted therapy, and immunotherapy, are available for patients with gastric cancer. Surgical resection is an effective way to improve the survival rate of these patients. Among the surgical options available, D2 radical gastrectomy has become the standard method for advanced gastric cancer. Since Goh et al. first reported laparoscopic radical gastrectomy for advanced gastric cancer in 2001, laparoscopic gastric cancer surgery has been developed rapidly and is used worldwide, especially in countries with a high incidence of gastric cancer, such as Japan, South Korea and China [4]. One of the unique advantages of laparoscopic surgery is its minimally invasive nature, but in view of the need for D2 lymph node dissection for patients with advanced gastric cancer, this operation is difficult and complex, so initial laparoscopic surgery is used only for the treatment of early-stage gastric cancer. After more than 10 years of research, large samples of multicentre clinical data have confirmed the safety, feasibility and effectiveness of laparoscopic radical gastrectomy for the treatment of early gastric cancer [5]. The latest Chinese Laparoscopic Gastrointestinal Surgery Study-01 (CLASS-01) findings state that “laparoscopic distal gastric cancer D2 radical resection performed by an experienced team is safe and feasible for the treatment of locally advanced gastric cancer” [6]. Upon reviewing the development of and advancements in gastric cancer surgery, we find that it has gradually changed from “standard and open surgical resection” to “individualized and accurate minimally invasive surgery” with further improvements to the safety of the operation and the quality of life of the patients postoperatively [7]. The new approach guided by the concept of laparoscopic minimally invasive surgery not only reduces the size of the surgical incision but also minimizes tissue trauma and maximizes functional preservation on the basis of radical oncology [8].

The concept of enhanced recovery after surgery (ERAS) was first proposed by Kehlet and is considered an important milestone in the development of surgery in recent years [9]. Its core goal is to adopt a series of optimized measures performed during the perioperative period on the basis of evidence-based medical findings to reduce the physiological and psychological stress of patients and to accelerate their recovery [10]. In contrast to traditional perioperative management, ERAS combines new techniques in anaesthesiology, pain, nutrition, psychology and surgery with traditional perioperative management by integrating medical interventions to accelerate the postoperative rehabilitation of surgical patients and ultimately improve their clinical outcome [11]. Our centre published the results from the first international randomized controlled trial (RCT) on the effect of ERAS on the short-term outcomes of postoperative patients with gastric cancer, proving that, compared with the traditional perioperative treatment regimen, the ERAS regimen is safe and feasible for perioperative gastric cancer patients. ERAS can reduce postoperative stress, shorten hospital stay, and improve patient quality of life, and it does not increase the incidence of postoperative complications [12]. Recent studies have shown that surgical stress can also affect the long-term oncological results of digestive tract tumours [13]. The mechanism underlying this effect may be immunosuppression as well as changes in the immune response, leading to a higher recurrence rate and more distant metastases. Surgical trauma can cause local and systemic inflammation, which can also result in the rapid growth of residual and micrometastatic diseases [14–16]. The ERAS pathway can reduce the systemic inflammatory response and facilitate early reversal of the human stress response, and it has been shown to significantly reduce the incidence of postoperative complications; thus, it has the potential to improve long-term oncology results. These results suggest that the application of the ERAS management pathway may not only improve short-term outcomes such as hospitalization days, postoperative complications, and mortality but also benefit tumour patients in terms of long-term survival. Ljungqvist et al. showed that ERAS pathway management played a positive role in the long-term survival of patients with colorectal cancer [17]. However, a prospective study investigating the effect of the ERAS pathway on the prognosis of gastric cancer has not yet been reported.

## Objectives

The purpose of this study is to investigate the impact of perioperative ERAS pathway management on the clinical safety and prognosis of patients undergoing laparoscopic-assisted distal gastric cancer radical surgery.

## Trial design

This experiment will include patients randomly divided into two groups – an experimental group and a control group – in a 1:1 ratio. The perioperative period of the experimental group will be managed according to the ERAS pathway and that of the control group will be managed according to the traditional management. After the patient was admitted to the hospital, imaging and haematological examinations will be performed; the risk assessments of nutrition risk screening 2002 (NRS2002), vein thromboembolism, American Society of Anesthesiologists will be administered; contraindications will be excluded; and laparoscopic-assisted radical resection of distal gastric cancer (D2, Billroth I, Billroth II, Roux-en-Y) will be performed under anaesthesia.

The trial will assess the clinical safety of the ERAS pathway and its impact on long-term survival. The effect of the ERAS pathway on inflammatory factors – leukocyte count, neutrophil percentage, C-reactive protein (CRP), procalcitonin, tumour necrosis factor-alpha (TNF-α) and interleukin-6 (IL-6) – will also be explored. The sample size is calculated according to survival rate, follow-up time, inferior value, grouping ratio, test efficiency, and loss of follow-up rate. A complete checklist of items according to SPIRIT (Standardized Protocol Items: Recommendations for Intervention Trials) (2013) is provided [18].

## Methods: Participants, interventions and outcomes

### Participant selection and randomization

**Fig. 1:**
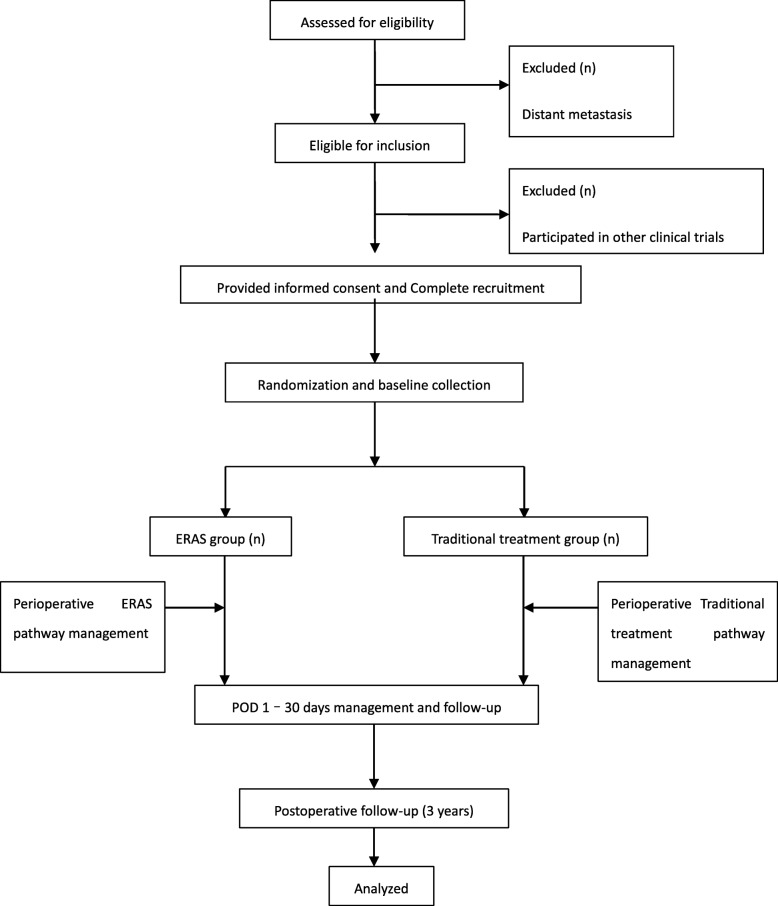
Flowchart of patient enrolment and randomization. *Abbreviations*: *ERAS* enhanced recovery after surgery, *POD* postoperative day.

Patients who have a diagnosed middle and lower gastric adenocarcinoma and who underwent laparoscopic-assisted radical resection of distal gastric cancer will be recruited from the 13 hospitals listed in Table 1. To achieve adequate enrolment, all surgeons in the gastrointestinal departments of the cooperating hospitals have been informed of this trial. Patients will be recruited for the study from the gastrointestinal surgery or gastrointestinal surgery outpatient clinic or by referral from the local affiliated hospital. The enrolment period is expected to be completed within 10 months from the beginning of recruitment. A total of 400 eligible patients will be selected and randomly (1:1) enrolled in the ERAS group and the traditional treatment group. Figure 1 shows the test group selection flowchart.

**Table 1:** The 13 participating surgical centres

Number	Centre	Department and investigator
1	Affiliated Hospital of Qingdao University	Gastrointestinal Surgery, Yanbing Zhou
2	Shandong Provincial Hospital	Gastrointestinal Surgery, Leping Li
3	Qilu Hospital of Shandong University	Gastrointestinal Surgery, Qingsi He
4	Qianfoshan Hospital of Shandong Province	Gastrointestinal Surgery, Lijian Xia
5	Second Hospital of Shandong University	Gastrointestinal Surgery, Yinlu Ding
6	Yantai Yuhuangding Hospital	Gastrointestinal Surgery, Lixin Jiang
7	Weihai Municipal Hospital	Gastrointestinal Surgery, Huanhu Zhang
8	Weifang People’s Hospital	Oncological Surgery, Yiran Shi
9	Dongying People’s Hospital	General Surgery, Hao Wang
10	Rizhao People’s Hospital	General Surgery, Xizeng Hui
11	Qingdao Municipal Hospital	Gastrointestinal Surgery, Weizheng Mao
12	Jining People’s Hospital	Gastrointestinal Surgery, Xianqun Chu
13	Weihai Central Hospital	Gastrointestinal Surgery, Xinjian Wang

For randomization, a central dynamic, stratified strategy is adopted. The randomization sequence is generated by using the Pocock–Simon minimization method in SAS version 9.3 (SAS Institute Inc., Cary, NC, USA) and stratified by participating site (13 hospitals) and surgical procedure (laparoscopic or robotic). Participating centres will submit the above information to the data centre at the Department of Gastrointestinal Surgery, Affiliated Hospital of Qingdao University, Qingdao, China, where central randomization will be performed. Information on treatment allocation is subsequently sent to each participating centre.

The inclusion criteria were as follows: (1) newly treated patients with no chemotherapy, radiotherapy or other anti-tumour treatment performed before the beginning of the clinical trial; (2) patients between 18 and 75 years of age; (3) patients at a clinical stage of advanced T (1–4a), N (0–3) or M0 scheduled to undergo radical resection of distal gastric cancer; (4) male or non-pregnant and lactating females; (5) patients with pathologically diagnosed gastric adenocarcinoma; (6) patients with Eastern Cooperative Oncology Group (ECOG) score 0–1; and (7) patients who voluntarily sign the informed consent form.

The exclusion criteria were as follows: (1) other malignant tumours within 5 years, (2) M1 disease found during the operation, (3) severe or uncontrolled medical diseases and infections found at the same time, (4) use of opioid analgesics or hormones within 7 days before the operation, (5) severe or uncontrollable mental illness, (6) any unstable condition or condition that may endanger the safety and compliance of the patient, and (7) participation and treatment with anti-cancer drugs in other clinical trials.

### Perioperative management

Before surgery, chest computed tomography (CT), total abdominal enhanced CT, and pelvic CT will be performed to confirm the size and location of the tumour, and distant organ metastasis will be excluded according to evaluations by two experienced radiologists. Upper abdominal CT angiography will be used to evaluate variation in the gastric blood supply of the patients, reduce the risk of intraoperative bleeding and guide lymph node dissection [19]. Echocardiography and pulmonary function tests will be used to evaluate the tolerance of cardiopulmonary function to laparoscopic surgery.

Laparoscopic-assisted radical resection of distal gastric cancer will be performed under general anaesthesia. During the operation, we will follow the basic principles of tumour treatment, master the appropriate scope of gastrectomy, perform fine lymph node dissection and gastrointestinal reconstruction, and record the amount of intraoperative infusion, blood loss, operation time, and use of any opioids or muscle relaxants.

After the operation, any adverse reactions that occur will be closely observed and actively treated. All drugs used will be recorded and described on the case report form (CRF). Laboratory examinations will be performed before the operation and 2 , 4 and 7 days after the operation. The measurements will include routine blood, liver and kidney function, electrolytes, procalcitonin, CRP, IL-6 and TNF-α. For patients with pathological stage II or above, 6–8 cycles of S-1 capsule combined with oxaliplatin adjuvant chemotherapy will be performed. Finally, oncology experts will choose the scheme and duration of treatment in accordance with the actual situation of the patients.

### Intervention protocols

Laparoscopic-assisted radical resection of distal gastric cancer will be performed by an experienced surgical team from the 13 centres listed in Table 1. Each of these centres performs at least 100 gastric cancer operations per year. Lymph node resection will be performed under laparoscopy, the main anastomosis will be performed with the assistance of a small incision, and the abdominal incision will be less than 8 cm. According to the research programme, the experimental group will actively carry out pre-rehabilitation before the operation, including lifestyle intervention, exercise advice, diet guidance, and health education (outpatient and hospitalization individualized condition consultation and answer). The specific interventions are shown in Table 2. However, target-oriented liquid management and early enteral nutrition (EN) after surgery require special attention. The goals of goal-directed therapy are to maintain central euvolemia while avoiding excess salt and water and a 24-h postoperative fluid balance on + 1 to 1.5 L. Intraoperative detection indicators are as follows: blood pressure, cardiac output, estimated blood loss, end-tidal carbon dioxide, and heart rate. The maintenance fluid flow rate is 1~4 mL kg^− 1^ h^− 1^ (predicted body weight), and large deviations from “zero balance” should be avoided [20]. The well-defined principles for oral intake in the ERAS groups are as follows: drinking a small amount of water and chewing xylitol on the day of operation; drinking 500~1000 mL water and chewing xylitol on postoperative day 1 (POD1); oral EN (mainly polypeptide) and chewing xylitol on POD2; oral EN (mainly integrin type) and chewing xylitol on POD3; oral EN, a small amount of semifluid and chewing xylitol on POD4; oral EN, semifluid (mainly) and chewing xylitol on POD5, but the patients in the traditional treatment group began sequential EN support treatment in accordance with the dietary pattern of the ERAS group after anal exhaust.

**Table 2:** Perioperative pathway management for gastric cancer

Programme components		ERAS group	Traditional treatment group
Preoperative	*Health education, exercise advice, dietary guidance	Yes	Yes
	*Organ function evaluation	Yes	Yes
	*Pre-rehabilitation treatment	Yes	No
	*MDT, clinical decision making	Yes	Yes
	*Preoperative nutritional assessment and intervention	Yes	Yes
	**Intestinal preparation**	EN No mechanical bowel preparation	No Traditional mechanical intestinal preparation
	*Preoperative fasting and abstinence from drinking	Fasting 6 h before the operation 2-h oral glucose infusion 200 mL	Fasting and drinking for 6 h before the operation
Intraoperative	*Intraoperative safety check (Checklist)	Yes	Yes
	**Local anaesthesia in the deep layers of the incision at the end of surgery**	Local anaesthesia (30 mL 0.25% bupivacaine)	No
	**Prevention of antibiotic use**	30 min before operation, operation time > 3 h, or more than one bleeding event ≥ 1000 mL	Application for 1–2 days
	*Surgical incision	Small midline (< 8 cm) incision at the upper abdomen	Small midline (< 8 cm) incision at the upper abdomen
	*Precision surgery	Laparoscopic or robotic surgery	Laparoscopic or robotic surgery
	*Anaesthesia mode	General anaesthesia combined with epidural anaesthesia^a^ (T7–T9)	General anaesthesia
	**Intraoperative heat**^**b**^**preservation**	Yes	Yes
Postoperative	**Urinary catheter**	Removal within 24 h	Routine indwelling catheter for 1–3 days after operation (until the patient is ambulatory and can urinate on his own)
	**Abdominal drainage tube**	Avoid placement or removal early after the operation as much as possible	Removal before discharge^c^
	**Gastric tube**	No use or removal ≤ 24 h	Retention for 1–3 days^d^
	*Early bedside activity	Start cautiously and plan your activities	2–3 days after operation
	*Postoperative analgesia	Multimodal analgesia^e^	Opioids^f^
	*Target-oriented liquid management	Yes	No
	**Prevention of deep venous thrombosis**	Basic prevention + physical prevention + drug prevention	Drug prevention
	*Early EN after operation	Sequential EN treatment after awakening from anaesthesia	Gradually start EN after anal exhaust

* Core provisions of perioperative enhanced recovery after surgery (ERAS) pathway management

*Abbreviations*: *EN* enteral nutrition, *MDT* multidisciplinary team, *NSAID* non-steroidal anti-inflammatory drug

^a^Dose/drug: 500 mg of ropivacaine + 400 mg of lidocaine and liquid intake rate of 2 mL/h

^b^Heat preservation measures: Pre-heated fluid replenishment, thermal blanket, heater

^c^Extubation indication: The drainage fluid is light red or clear, with a volume of less than 20 mL, and pancreatic amylase is negative for 24 h

^d^Criteria for the removal of nasogastric tube: Recovery of intestinal peristalsis, anal exhaust and oral intake of clear fluids

^e^Multimodal analgesia: postoperative day 1~2 (POD1~2) patient controlled epidural analgesia (lidocaine + ropivacaine); POD3~5, 0.65 g of regular oral paracetamol every 8 h (q8h); when the visual analogue scale ≥ 4, 50 mg of flurbiprofen is injected intravenously

^f^Opioids: POD1~2, 50 mg of tramadol q8h; when the visual analogue scale ≥ 4, 50 mg of tramadol is injected intravenously (dose ≤ 400 mg/d)

### Study endpoints

The main endpoint is the comparison of 3-year overall survival (OS) and disease-free survival (DFS) between the ERAS pathway group and the traditional treatment group. The secondary endpoints are the total incidence of postoperative complications, incidence of major complications, 30-day re-hospitalization rate, 30-day mortality rate, hospitalization days and hospitalization costs as well as other short-term clinical outcomes. The exploratory results are changes in inflammatory indexes (i.e., leukocytes, neutrophil percentage, CRP, procalcitonin, TNF-α and IL-6).

### Data collection and management

Once written informed consent is provided, the clinical researchers will collect baseline data such as age, sex, body mass index, and complications. The laboratory indexes—routine blood, liver and kidney function, electrolyte, carcinoembryonic antigen, carbohydrate antigen 199 (CA199), CA724, CA242, alpha fetoprotein (AFP), hepatitis, human immunodeficiency virus, syphilis, blood coagulation routine, and blood type—will also be assessed and recorded before and during hospitalization. The designated surgeon will record the details of the procedure, such as the surgical approach, the location of the tumour, lymph node metastasis, and pathological stage.

Starting on day 1 after the operation, the clinical observation data (e.g., extubation time, food intake, activity, anastomotic leakage, first exhaust and defecation time, postoperative hospital stay, and complications) will be recorded daily by nurses to evaluate postoperative recovery. Clinicians will be responsible for patient management and will not be involved in data collection.

All relevant information for each patient should be recorded in the CRF in a timely and accurate manner by trained and independent research staff. If there are any errors in the CRF, the investigator will correct them immediately. When revising raw data, the investigator must sign their name and the date. All data will be acquired only by study investigators who have signed a confidential disclosure agreement. In this clinical study, any collected information that could be used to disclose an individual’s identity will not be released or disclosed at will without consent, except in special circumstances as required by law. No research publications using these data, including journal literature, papers or research briefs, will use any identifying patient information. The ethics committee of the Affiliated Hospital of Qingdao University will be responsible for ensuring that the rights and well-being of patients are protected and for maintaining compliance with the currently approved protocol, data collection, statistical analysis, and anonymity in publications.

### Discharge criteria

The criteria for discharge are as follows: (1) postoperative pain score with oral analgesics controlled well (visual analogue scale score below 4), (2) oral semifluid food without intravenous rehydration, (3) satisfactory exercise regimen (6 h a day or up to preoperative level), (4) adequate out-of-hospital care, (5) voluntary discharge of the patient, and (6) no surgical complications, such as fever, abdominal pain, or infection. In addition, the contact information and address of each patient will be confirmed before discharge. Follow-up will be conducted by telephone within 24 h after discharge, and the focus will be on dietary tolerance, pain, defecation and any discomfort.

### Follow-up

After the operation, a special follow-up team will be responsible for performing patient follow-up, and the first outpatient review will begin at 3 weeks after the end of treatment.

**Fig. 2:**
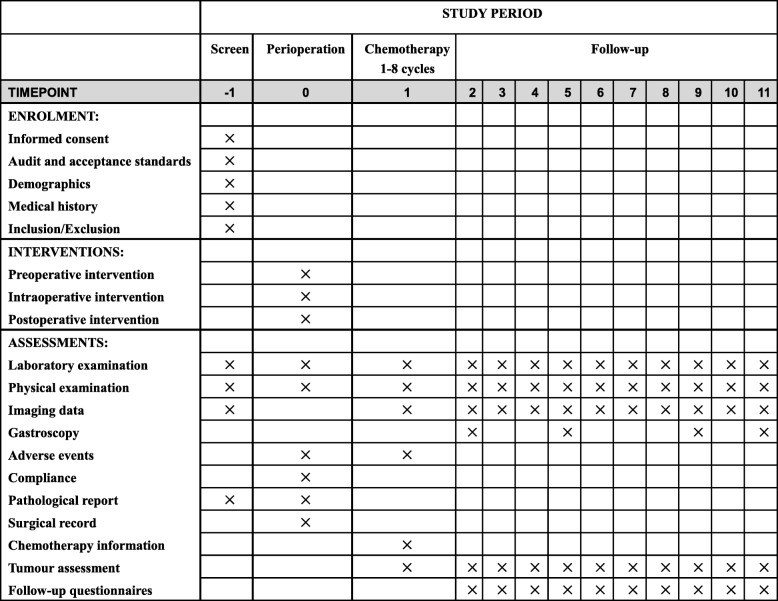
Flowchart of the multicentre clinical trial for the schedule of enrolment, interventions and assessments. The symbol “×” indicates the project that must be completed during the research phase; −1, 2 weeks before operation; 0, perioperation; 1, adjuvant chemotherapy time; follow-up 2~11 corresponding time points are the following: 2, 3 months after operation; 3, 6 months after operation; 4, 9 months after operation; 5, 12 months after operation; 6, 15 months after operation; 7, 18 months after operation; 8, 21 months after operation; 9, 24 months after operation; 10, 30 months after operation; 11, 36 months after operation.

From 0~2 years after the operation, follow-up every 3 months will include a routine blood examination, an analysis of biochemical markers and digestive tract tumour indicators, and imaging examinations. In addition, endoscopic examinations will be performed once a year. At each follow-up, the adjuvant treatment, postoperative recovery, and short-term and long-term side effects will be assessed, as shown in Fig. 2. From 2~3 years after the operation, follow-up will occur every 6 months, as outlined above.

### Statistical analyses

Classification variables will be analysed by the chi-squared test or Fisher’s exact test, and continuous variables will be analysed by the independent *t* test. DFS will be defined as the time from surgery to death or recurrence of gastric cancer, whichever occurs first. The Kaplan–Meier method will be used to generate survival curves, and the log-rank will be applied to compare the differences between survival curves. The hazard ratio and 95% confidence interval will be calculated with the Cox regression model. Variables will be selected for inclusion in the final multivariable model using a stepwise method, and significance levels of 0.25 and 0.15 will be employed as the criteria for inclusion and retention. A *P* value of less than 0.05 will be considered to indicate statistical significance. Data analysis will be performed by using SPSS® software package version 22.0 (SPSS Inc., Chicago, IL, USA).

### Sample size estimate

This study adopts the design of a non-inferiority test, and the calculation of sample size is based on the following historical data and assumptions. Previous studies have shown that the overall 3-year survival rate for patients with gastric cancer is about 50% [5]. The centre followed patients who underwent radical resection of gastric cancer under the management of ERAS from 2011 to 2014, and the 3-year survival rate was about 65% [21]. Given that patient selection will require 10 months, the median follow-up time should be about 3 years, and therefore the non-inferiority threshold is set to 1.33, according to a 1:1 random ratio. Given a significance level of α = 0.05 (bilateral) and test efficiency of 1 − β = 80%, the withdrawal rate of either branch group should be 10% and thus the total sample requires at least 400 patients (200 in the test group and 200 in the control group).

### Interim analyses and trial termination

This clinical trial project plans to recruit 400 patients and conduct an interim data analysis, faithfully reflecting changes in their condition during and after the operation, at the point in which about 200 patients have been enrolled. To improve the trial further, we have established a data monitoring committee that consists of surgical experts, statistics experts and ethics experts, independent of the clinical research team of the project, to weigh the effectiveness and safety comprehensively at the midpoint of the clinical trial according to the data accumulated to date and then make important decisions regarding whether to “continue the trial”, “continue the trial after adjusting the protocol” or “terminate the trial”. The results of the interim analysis will be released to all investigators.

### Strengths and limitations of this study

The feasibility of ERAS pathway management in improving long-term prognosis has not yet been determined in a prospective RCT. This trial will be the first multicentre randomized controlled clinical trial to evaluate the impact of perioperative ERAS pathway management on the short-term clinical outcomes and long-term prognoses of patients undergoing laparoscopic-assisted radical resection for distal gastric cancer. Jieshou Li, an academician affiliated with the Chinese Academy of Sciences, introduced this concept in 2007. Our centre began to explore ERAS pathway management for patients with gastric cancer during the same year and published RCT research findings on perioperative ERAS management for gastric cancer in 2010 [12]. Our team has accumulated rich experience in perioperative management for gastric cancer to ensure the safety of patients and enhance their recovery. At the same time, our cooperating centres are all members of the gastric cancer ERAS group (each with an annual operation volume of more than 100 cases), have undertaken national clinical projects, and have performed strong clinical research. The primary outcome of this study is the comparison of 3-year OS and DFS between the two groups. The ERAS team will be required to record the data in a timely and accurate manner and to enhance postoperative follow-up in order to avoid loss to follow-up. To this end, we specifically established a data inspection committee and a special follow-up team to ensure the timeliness, validity and authenticity of the clinical data.

In this study, it is expected that owing to individual differences, patient compliance, medical factors and other reasons, the ERAS team will experience some difficulty in completely implementing all interventions in the protocol. We will integrate the elements involved in the clinical pathway. To ensure recovery and reduce hospitalization in the days after gastric surgery, we will pay particular attention to the “key components” of the ERAS programme, namely six basic elements: (1) preoperative patient information and education, (2) preoperative pre-rehabilitation, (3) thoracic epidural anaesthesia combined with multimodal analgesia, (4) target-oriented liquid management, (5) no nasogastric tube, and (6) early oral feeding and mobilization.

## Discussion

The core of the ERAS concept is to use perioperative optimization measures based on evidence-based medicine to reduce surgical trauma and the stress response and to promote postoperative recovery. This concept has subverted the thinking and principles of perioperative management formed over the past hundred years and has created a new concept of rehabilitation. The ERAS pathway not only can improve the early clinical outcomes of patients with gastric cancer but also (it is hoped) can improve the survival rate of patients. This study is a prospective, multicentre, open, randomized controlled clinical trial that aims to provide important evidence support to achieve this goal.

In recent years, many international large-scale gastric surgery centres have begun to explore the ERAS pathway for gastric cancer. The application of the ERAS pathway in the perioperative management of gastric surgery has been repeatedly proven to be able to reduce postoperative complications, shorten postoperative hospital stays, relieve postoperative pain and reduce total hospitalization costs [11, 22]. Unfortunately, the ERAS pathway has limited research on improving the survival of patients with gastric cancer. Current observational studies have shown a significant association between ERAS compliance and colorectal cancer survival. In patients with more than 70% adherence to ERAS interventions, the risk of 5-year cancer-specific death was lowered by 42%, hazard ratio 0.58 (0.39–0.88, Cox regression), compared with all other patients (≤70% adherence) [17]. At present, one mechanism of the ERAS pathway to improve this result is to reduce the surgical stress response [9]. Some studies have shown that perioperative stress not only can affect tumour recurrence [23, 24] but also can stimulate dormant micrometastases and minimal residual cancer [25–27]. In addition, under the ERAS management mode, the immune function after operation can be better preserved. Studies have shown that surgery is related to short-term immunosuppression after surgery [27]. Pro-inflammatory cytokines released after surgery, such as TNF-α and transforming growth factor-beta (TGF-β), have also been shown to stimulate tumour cell adhesion [28, 29]. Patients managed by the ERAS pathway show better preservation of cell-mediated immunity and immune function and a lower stress response, thereby inhibiting tumour recurrence and metastasis and prolonging patient survival [30, 31].

Overall, the ERAS pathway has been proven to be a safe and effective perioperative management model in the recent literature. In particular, the ERAS pathway has shown promising results in improving the survival of patients with gastric cancer. Confirmation of these results is essential by means of RCTs.

## Trial status

The enrolment of this study was ongoing at the time of manuscript submission. The protocol version is 1.1, GISSG18–01, 10 March 2019. The trial will be ongoing from 10 April 2019 to 30 June 2020.

## Data Availability

Data from this randomized controlled study were unavailable at the time of publication. Individual participant data are available upon request.

## Abbreviations

CRF: Case report form
CRP: C-reactive protein
CT: Computed tomography
DFS: Disease-free survival
EN: Enteral nutrition
ERAS: Enhanced recovery after surgery
IL-6: Interleukin-6
OS: Overall survival
POD: Postoperative day
RCT: Randomized controlled trial
TNF-α: Tumour necrosis factor-alpha
